# 3DScapeCS: application of three dimensional, parallel, dynamic network visualization in Cytoscape

**DOI:** 10.1186/1471-2105-14-322

**Published:** 2013-11-14

**Authors:** Qi Wang, Biao Tang, Lifu Song, Biao Ren, Qun Liang, Feng Xie, Ying Zhuo, Xueting Liu, Lixin Zhang

**Affiliations:** 1 Chinese Academy of Sciences Key Laboratory of Pathogenic Microbiology and Immunology, Institute of Microbiology, Chinese Academy of Sciences, Beijing 100190, People’s Republic of China; 2 State Key Laboratory of Genetic Engineering, Department of Microbiology, School of Life Sciences, Fudan University, Shanghai 200433, People’s Republic of China; 3 Institute of Bioprocess and Biosystems Engineering, Hamburg University of Technology, Hamburg, Germany; 4 University of Chinese Academy of Sciences, Beijing 100049, People’s Republic of China

## Abstract

**Background:**

The exponential growth of gigantic biological data from various sources, such as protein-protein interaction (PPI), genome sequences scaffolding, Mass spectrometry (MS) molecular networking and metabolic flux, demands an efficient way for better visualization and interpretation beyond the conventional, two-dimensional visualization tools.

**Results:**

We developed a 3D Cytoscape Client/Server (3DScapeCS) plugin, which adopted Cytoscape in interpreting different types of data, and UbiGraph for three-dimensional visualization. The extra dimension is useful in accommodating, visualizing, and distinguishing large-scale networks with multiple crossed connections in five case studies.

**Conclusions:**

Evaluation on several experimental data using 3DScapeCS and its special features, including multilevel graph layout, time-course data animation, and parallel visualization has proven its usefulness in visualizing complex data and help to make insightful conclusions.

## Background

Cytoscape [1] is a free open source platform providing biological network analysis and two-dimensional (2D) visualization for biologists. With more than 172 registered plugins contributed by the community, it is very versatile in network applications, such as network importing, network integrating, inference customization, literature mining, topological clustering, functional enrichment, network comparison, and programmatic access [2]. However, it lacks the capability of displaying the large-scale networks in three-dimensions (3D) or beyond. With the exponential growth of gigantic biological data deposited in the public domain, the extra space visualization would offer more flexibility for layered representation [3] and heterogeneous data visualization [4]. Although a few applications are available for 3D network visualization, such as BioLayout [5], Wilmascope 3D [6], Arena3D [7], FORG3D [8], the lack of community support has hindered their widespread use. Tulip [9] features 3D display and a variety of plugins, but it is far less versatile as Cytoscape in biological studies. Moreover, some of them suffer performance drawbacks when displaying large networks (Table 1). Although there are Cytoscape’s 3D plugins using Processing library [10] and JOGL [11], they are still under development. There is also an approach using RCytoscape [12] and RGL to create 3D visualization for Cytoscape, but it is not well developed in terms of its layout performance and ease of use. Although the release of Cytoscape 3.0 [13] claims that through its OSGi engine, it enables user to switch renderers in Cytoscape. However, graph manipulation and layout algorithms are both required to be changed while switching 2D to 3D, which means excessive override of Cytoscape core modules. Taking into consideration of the difficulties faced in implementation, adopting a light-weighted external renderer is a better choice. Moreover, C++ implementation is often superior in performance than Java when it comes to layout large graph with more than ten thousands nodes. Therefore we choose UbiGraph [14], which is a 3D visualization tool supporting up to eight POSIX threads and multilevel force-directed graph layout of high performance and aesthetic pleasure. We developed 3DScapeCS, a Cytoscape plugin providing three-dimensional, dynamic, parallel network visualization for Cytoscape in client–server (C/S) architecture (in Additional files 1 and 2). Despite criticism that developers favour in building 3D visualization tools rather than analyzing using such tools has arisen within the bioinformatics community [15]. 3DScapeCS has undergone extensive testing among molecular chemistry, genomics and proteomics studies, which showcased as the following (in Additional file 3).

**Table 1:** A comparison between available 3D network visualization tools

**Program name**	**File format**	**Operating system**	**Programming language**	**Source code availability**	**Time course data support**	**Layout algorithm**	Performance*
Arena3D [7]	Arena3D	Platform-Independent	Java	yes	yes	Layered	2,000 nodes, 10,000 edges
BioLayout	SIF, XML, TXT, GraphML, Matrix, Expression	Platform-Independent	Java	yes	yes	Fruchterman-Rheingold	500 nodes, 2,500 edges
Express 3D [5]							
FORG3D [8]	FORG3D	Windows, Linux and Mac OS X	C++	yes	no	Force-directed	4,000 nodes, 40,000 edges
Vaa3D [4]	TIFF, SWC, CSV	Windows, Linux and Mac OS X	C++	yes	yes	Location based	2,500 textured nodes and edges
Wilmascope 3D [6]	Wilmascope, GML	Platform-Independent	Java	yes	yes	Force-directed, etc.	2,000 nodes, 5,000 edges
3DScapeCS	any format supported by Cytoscape	Platform-Independent	Java	yes	yes	Multilevel Force-directed	10,000 nodes, 250,000 edges

*Approximate network size measured with minimum frames per second (fps) which enables smooth manipulations (drag, rotate, etc.) on an eight core Intel Xeon 2.13 GHz workstation with an on-board Matrox G200 graphics card.

## Implementation

### Visualization of three-dimensional networks

**Figure 1:**
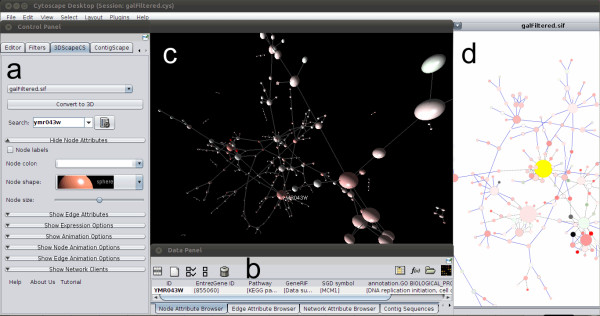
**The 3DscapeCS interface.** Running 3DscapeCS in Cytoscape. **(a)** 3DScapeCS control panel; **(b)** Selected nodes/edges are listed in Attribute Browser; **(c)** Test data converted into 3D view using UbiGraph; **(d)** Corresponding 2D view in Cytoscape.

3DScapeCS converts an existed Cytoscape network to a 3D view as shown in Figure 1a. The conversion can retain almost all settings such as the colours, sizes, and shapes of all nodes as similar as those from the existing 2D view. The users can easily manipulate the 3D view by using mouse. Operations including rotation and zooming of the display have been implemented in UbiGraph. We have implemented network communication between UbiGraph and Cytoscape as well. On double-clicking a node in UbiGraph, its attributes will be shown in the Node/Edge Attribute Browser panel in Cytoscape (Figure 1b). A searching box is provided for user to search the nodes/edges by corresponding identifiers. Whenever the search succeeds, the node matched will be highlighted with a label in the 3D view (Additional file 4).

### Three-dimensional layout

The layout algorithms are required to generate meaningful and aesthetic drawings of biological networks. The layout algorithm built in UbiGraph is force-directed based. It acts by balancing the repulsive force between nodes and attractive force from the edge. After several rounds of movement and annealing, it generates aesthetically pleasing graph layouts with all edges of nearly equal length. The force-directed layout algorithm implemented in Cytoscape has a complexity of O(N^3^) and low-quality local drawing [16], it is only suitable for networks containing up to several hundred nodes. Other global force-directed algorithms such as Fruchterman-Reingold [17] experience the same bottleneck. Therefore local force-directed algorithms such as Fast Multipole Multilevel Method (FM3) [18] and Multilevel layout algorithm [14] have been developed. Hereby we adopted the multilevel layout algorithm in UbiGraph for larger networks. In this approach, the network is partitioned into sub-networks. Only forces within the scope of sub-networks are calculated, so that the final graph layout can be obtained in O(N*log*N) runtime. Therefore it will greatly reduce the duration required to complete three-dimensional layout, which is significantly helpful in visualization of large-scale networks (Figure 1c).

### Time-course data animation

**Figure 2:**
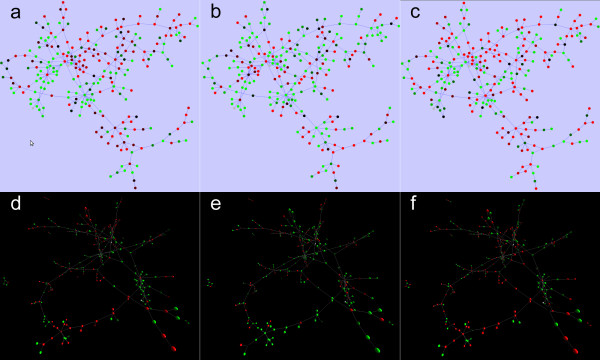
**Visualization of expression data.** Comparing 2D and 3D expression data visualization using VistaClara and 3DScapeCS using VistaClara heat map scheme. **(a,d)** gal1RGexp, **(b,e)** gal4RGexp, **(c,f)** gal80Rexp.

**Figure 3:**
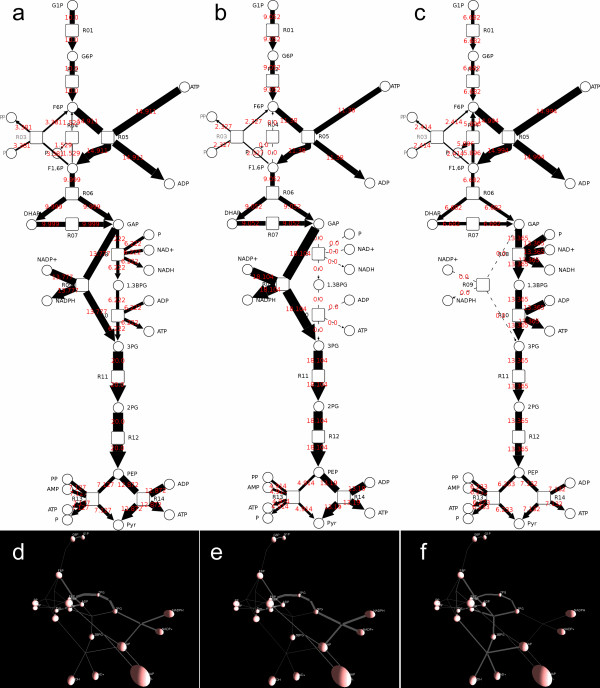
**Visualization of metabolic flux data in a substrate-product network.** Metabolic flux simulation in FluxMap **(a-c)** and 3DScapeCS **(d-f)**, a-f represents different genotypes. **(a,d)** WT, **(b,e)** “KO R08”, **(c,f)** “KO R09”.

**Figure 4:**
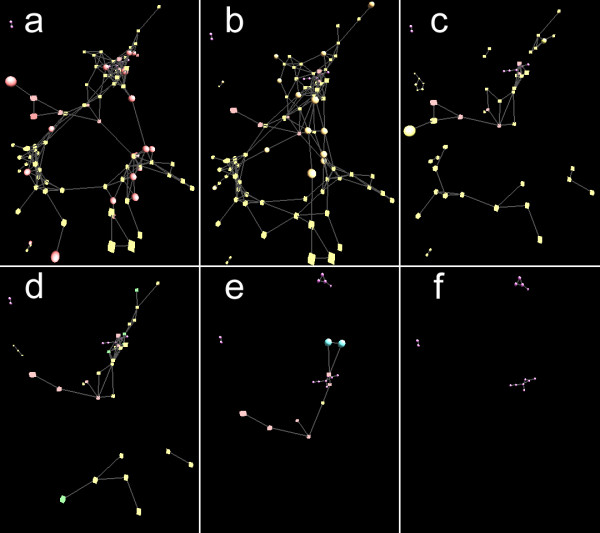
**Mass spectrometry (MS) motion network with six conditions. a-e** are snapshots of the motion network at different dose treatments using avermectin. **f** was treated with DMSO, which served as a negative control.

Cytoscape supports the mapping of gene expression data to node color, label, and border thickness. With VistaClara [19] or SpotXplore [20] plugin installed, the user can distinguish expression intensities from a heat map themed node colour scheme. VistaClara even creates an animated view of the network for displaying the changes over time in a series of experiments. In our implementation, users are able to explore expression data using VistaClara’s heat map scheme or user-defined color scheme (Figure 2). Moreover, time-course data can be attributed to node sizes or edge thickness, making it possible for visualizing various data types, such as data from metabolic flux analysis, with thicker edge representing larger flux, and vice versa (Figure 3). We also implemented motion network in 3DScapeCS, made it possible to study the changes over time or different conditions, such as mass spectral molecular networks (Figure 4).

### Parallel visualization

3DScapeCS provides an approach for visualizing one network from multiple aspects by adopting parallel visualization. The Cytoscape network view can be simultaneously rendered in UbiGraph on multiple computer clients through network communication. Different clients can be set to visualize different aspects of the graph, either a reversal of perspective, or another time-point from a time-course animation. Unlike CytoscapeRPC [21], which use XML-RPC to modify networks in Cytoscape from clients, UbiGraph clients serve as XML-RPC servers in 3DScapeCS architecture. Therefore any changes made to the network view in Cytoscape, either change the size/colour of a node, or add/delete a node, can be reflected on all clients by synchronizing the network data between UbiGraph clients and Cytoscape.

## Results

### Genome sequences scaffolding visualization

**Figure 5:**
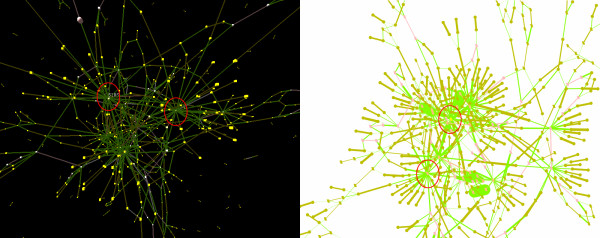
**Visualization of contig relationships.** Comparing 2D and 3D contig relationships visualization generated using ContigScape. Red circles show 27 connections with 5' end of contig No. 510 and 30 connections with 3' end of contig No. 510 in 3DScapeCS (left) and Cytoscape (right), respectively.

Next-generation sequencing (NGS) and third-generation sequencing technologies such as PacBio SMRT greatly facilitated whole genome sequencing. However, complex genomic structures that cause sequence bias even with high genomic coverage and repeated sequences that may cause gaps in assembly still hamper gap closure. Determining the relationships between contigs or scaffolds is therefore very important. Showing straightforward graph-based relationships of contigs in Cytoscape rather than tables is more intuitive, and will help planning for further PCR validation. ContigScape [22] is a Cytoscape plugin for helping visualizing genome scaffolds. However, complex contig relationships in highly repetitive regions are hard to resolve since they have many intersections. By converting such network into three-dimensional graph, the relationships between contigs become easier to be identified. In the project of *Ralstonia* sp. genome scaffolding [22], three repetitive contigs have several connections which are hard to be distinguished, while nodes are well dispersed and their relationships with other contigs are much clearer in corresponding 3D view (Figure 5).

### Dynamic visualization of metabolic flux data

**Figure 6:**
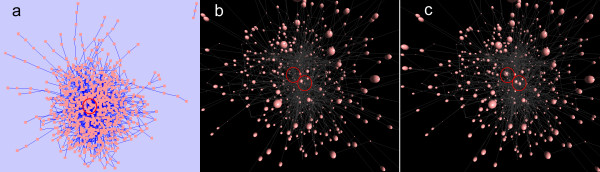
**Visualization of metabolic flux data in a reaction network.** Metabolic flux data for *E.coli* metabolism are visualized in a reaction network. **(a)***E.coli* metabolism network in Cytoscape. **(b)***E.coli* metabolism network in 3DScapeCS. **(c)***E.coli* metabolism network with simulated data. Each node represents a reaction, with its size corresponding to the reaction rate. The changed reaction found to compare are highlighted with red circles.

Flux balance analysis (FBA) is a powerful tool in simulating metabolism in genome scale reconstructions of metabolic networks [23]. Dynamic exploration of flux data will give user an intuitive guidance on metabolic changes. In 3DscapeCS, metabolic flux data visualization can either be reflected in node size (reaction network), or edge thickness (substrate-product network). Here we use *E. coli*’s energy metabolism model [24]. To reduce network complexity, we only show relationship with flux distribution greater than zero. Node sizes are rendered according to their flux distribution, so that reactions with greater flux can be easily identified (Additional file 5). We compare the reaction network between the initial state (Figure 6b) and the simulated state (Figure 6c). The reactions with changed rate are easier to be identified in the 3D view due to the nodes in the centre of the network are dispersed in the 3D space, while the nodes in the 2D view are overlaid with each other. The evaluations with substrate-product network were carried out between 3DScapeCS and FluxMap [25], which is a VANTED [26] add-on for the visual exploration of metabolic flux (Additional file 6). Although it features time-point simulation or conditions iteration in flux visualization, it lacks the “auto-play” functionality as 3DScapeCS does. Simulation of flux knockout example of glycolysis pathway in FluxMap example package shows that 3DScapeCS is useful in showing metabolic flux changes between different genotypes (Figure 3).

### Mass Spectrometry (MS) molecular network visualization

Molecular network has been used for the analysis of MS/MS data and provide a new perspective to study microbial natural products [27]. However, an efficient approach needs attention to interpret the similarities or differences between several conditions in biological experiments. Nguyen D. *et al.* has applied Cytoscape to visualize MS network generated from several MS experiments [28]. Here we present an example of the application 3DScapeCS in monitoring the changes of metabolomics of the human pathogen *C. albicans* after the treatments using different doses of avermetin, a potent antifungal natural product (Ren *et al*., unpublished data). In this experiment, we treated *C. albicans* with 400 (Figure 4a), 100 (Figure 4b), 25 (Figure 4c), 6.25 (Figure 4d), and 1.5625 (Figure 4e) μg/mL of avermectin, respectively. Meanwhile, the pathogen treated with DMSO was used as negative control (Figure 4f). The monitoring of the metabolites would help in identifying the pathway affected by avermectin and subsequently interprets the medicinal mechanism of avermectin. A motion graph varies in topology enables dynamic exploration of molecule changes at different conditions. This example is included in a saved Cytoscape session named MS.cys (in Additional file 3). User needs to export the session into SIF-format network and the filenames in node attributes into Cytoscape node attributes file. Then use separate.pl to divide the SIF-format network into several sub-networks according to their filename attributes provided. The generated SIF -format networks from A to F are at different dose treatments. Using “Order Networks”, user makes them a motion network (Figure 4). The topological changes are useful in distinguishing different molecules produced at different dose treatments (Additional file 7). The parallel visualization on multiple monitors and animation iterating all the networks also facilitate more comprehensive comparison. As a result, the avermectin treated cells produced more molecules compared with negative control (Figure 4f), and the molecules were gradually reduced along with the dose decreased, indicating that avermectin affected some pathways of *C. albicans* to produce or accumulate different molecules. Further identification of the different molecules from the node can help us to find the targeted pathway of avermectin.

### Detecting bubbles in De Bruijn graph

**Figure 7:**
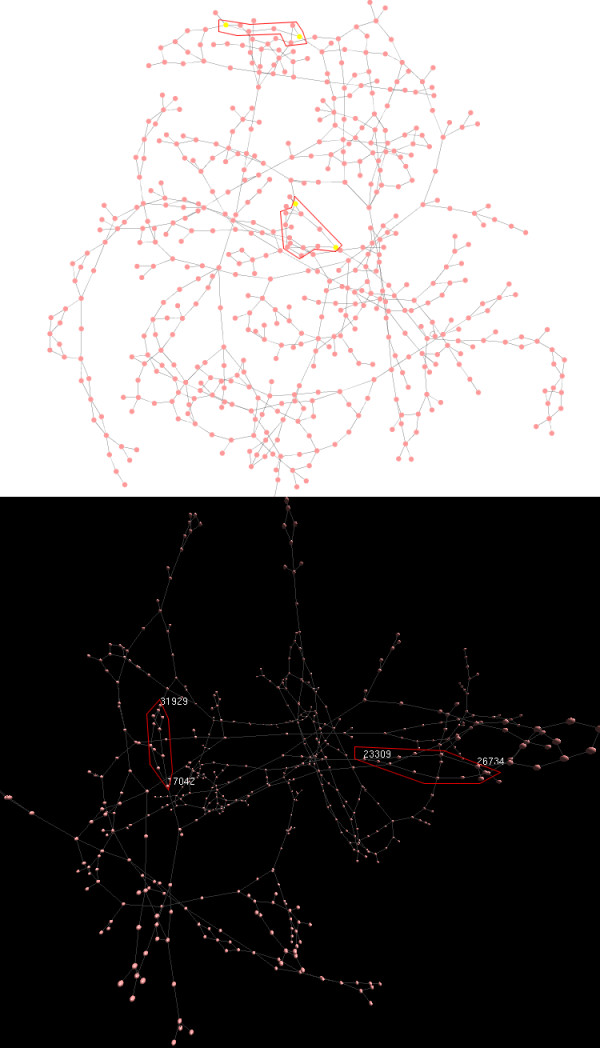
**Visualization of de Bruijn graphs converted from velvet.** Comparing 2D and 3D de Bruijn graph created from LastGraph file generated by Velvet. The bubbles found to compare are highlighted with red ellipse. Bubbles are not clear due to they were overlaid with other nodes or edges (upper) in Cytoscape. They become clear when rotated to a different perspective in 3DScapeCS (lower).

De Bruijn graphs [29] are very common among Next-generation sequencing tools such as Velvet [30], ALLPATHS [31], ABySS [32], Ray [33] and SOAPdenovo [34]. Assembly programs indexes reads in short words (*k*-mers). Each node represents a series of overlapped *k*-mers in de Bruijn graph, while arc represents their connection. Paths traversing the graph are joined into contigs. Due to the existence of biological variable and erroneous *k*-mers, de Bruijn graph often forms bubbles, which is defined as several similar paths sharing the same start node and end node [30]. Bubbles are hard to resolve by *de novo* assemblers, and therefore extension often ends up with an early termination in paths containing bubbles. In order to distinguish such termination from sequencing gaps, it is important in assessing the de Bruijn structure and detecting the bubbles. Here we evaluated a *de novo* assembly graph of *Streptomyces avermitilis* (Zhuo *et al*., unpublished data), which was assembled using Velvet v1.2. We converted the LastGraph produced by Velvet into SIF-format network with 1,891 nodes and 1,921 edges using a Perl script debruijn.pl (in Additional file 3). While the two-dimensional network visualization failed to show the bubbles between nodes after force-directed layout in Cytoscape, the three-dimensional visualization indicates there are three bubbles in the highlighted regions (Figure 7).

## Discussion

Three-dimensional visualization provides novel insights for Cytoscape. With time-course, colour, size, shape customization, 3DScapeCS can support up to seven dimensional data visualization, making it useful in presenting large-scale complex data. Although external renderers such as UbiGraph can add 3D functionalities to Cytoscape, the operations of network are limited in UbiGraph. For example, user can neither drag vertices or edges in the graph to set locations, nor set the parameters of the multilevel layout algorithm, which make it less effective in heterogeneous 3D data visualization. Therefore, in the future, a more powerful renderer featured with more layouts and subcellular localization networks, such as neuronal networks, should be implemented. Moreover, mobile devices such as iPad or Android Pad, which can be manipulated using fingers and gestures, are more suitable to manipulate 3D view. So it is also desirable to implement renderers on those platforms for wireless parallel visualization.

## Conclusions

We have integrated UbiGraph and Cytoscape in 3DScapeCS. The 3D perspective not only guarantees user a greater experience in visualization, but also offers more insight into Cytoscape networks. Parallel motion graph is useful in visualizing data obtained with different conditions, as well as different aspects in a single experiment. Such functionalities give full play to their strength on a three-dimensional platform. Therefore 3DScapeCS has more advantages in large and/or complex network visualization as the study cases presented in previous paragraphs.

## Availability and requirements

**Project name**: 3DScapeCS

**Project home page**: http://scape3d.sourceforge.net/

**Operating system(s)**: Platform-independent.

**Programming language**: Java

**Other requirements**: Cytoscape v2.8, UbiGraph needs to be started as its renderer.

**License**: Lesser General Public License (LGPL).

**Any restrictions to use by non-academics**: None.

## Competing interests

The authors declare that they have no competing interests.

## Authors’ contributions

QW conceived, designed and implemented the software and the case studies, tested the software and drafted the manuscript. BT, LFS and XTL tested the software, suggested modifications, performed the case studies, and contributed in drafting the manuscript. BR, QL, FX, YZ and LXZ contributed in drafting the manuscript. LXZ also supervised this project as a whole. All authors have read, revised and approved the manuscript.

## Supplementary Material

Additional file 1 **The 3DscapeCS Program File.** Put 3DscapeCS.jar into cytoscape/plugins/ folder. Click here for file

Additional file 2 The 3DscapeCS User Manual. Click here for file

Additional file 3 **The 3DscapeCS Case Study Data Unpack the zip package using WinZip, WinRAR, 7-zip or unzip command.** See REAME.txt inside the package on how to use the test data. Click here for file

Additional file 4 The 3DscapeCS Movie – Basic Operation and Expression Data Visualization. Click here for file

Additional file 5 The 3DscapeCS Movie – Reaction Network Motion Visualization. Click here for file

Additional file 6 The 3DscapeCS Movie – Substrate-product Network Motion Visualization. Click here for file

Additional file 7 The 3DscapeCS Movie – Motion Network Visualization of MS Experiments. Click here for file
