# Supplementary material for: 3DScapeCS: application of three dimensional, parallel, dynamic network visualization in Cytoscape
Source: BMC Bioinformatics. 2013 Nov 14;14:322. doi: 10.1186/1471-2105-14-322 (PMC3835703; doi:10.1186/1471-2105-14-322)
Supplement: Additional file 2 — The 3DscapeCS User Manual. [file 1471-2105-14-322-S2.pdf]

3DScapeCS User Manual

Version 0.7
Aug 31, 2013

Introduction

Thank you for choosing 3DScapeCS, which provides three dimensional, multi-desktop, dynamic network visualization in Cytoscape. You can get the latest version from http://sourceforge.net/projects/scape3d.

System Requirements

3DScapeCS runs on any platform with Cytoscape installed. However, 3DScapeCS requires Ubigraph, which can only run on Linux or MacOSX. You can download Ubigraph from http://www.ubietylab.net/ubigraph/

Installation Guides

Put 3DScapeCS.jar into Cytoscape/plugin directory. Ubigraph needs to be started outside Cytoscape, so check Ubigraph requirements for your system:

Ubuntu: sudo apt-get install libglut* or sudo apt-get install glut3*.

CentOS or Redhat: freeglut3 or equivalent.

MacOSX: nothing required.

Usage

Ubigraph_server should be started first before you use 3DScapeCS. Once Cytoscape finished loading, 3DScapeCS will be found in the left tab of Cytoscape panel. Try import a network in Cytoscape and select its label in the drop list, then use **"Convert to 3D"** button to create a three dimensional view in 3DScapeCS. The 3D network are rendered in Ubigraph_server window after the conversion is completed.

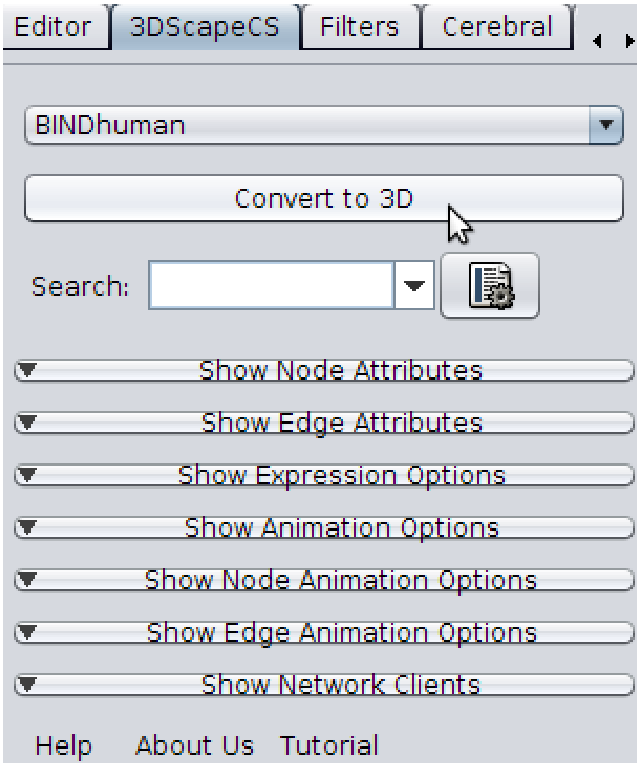

The node/edge are converted with styles created according to their shape, color, size and label in their Cytoscape network view. However not all the shapes in Cytoscape can be matched in Ubigraph, so the node shapes are converted according to the following rules:

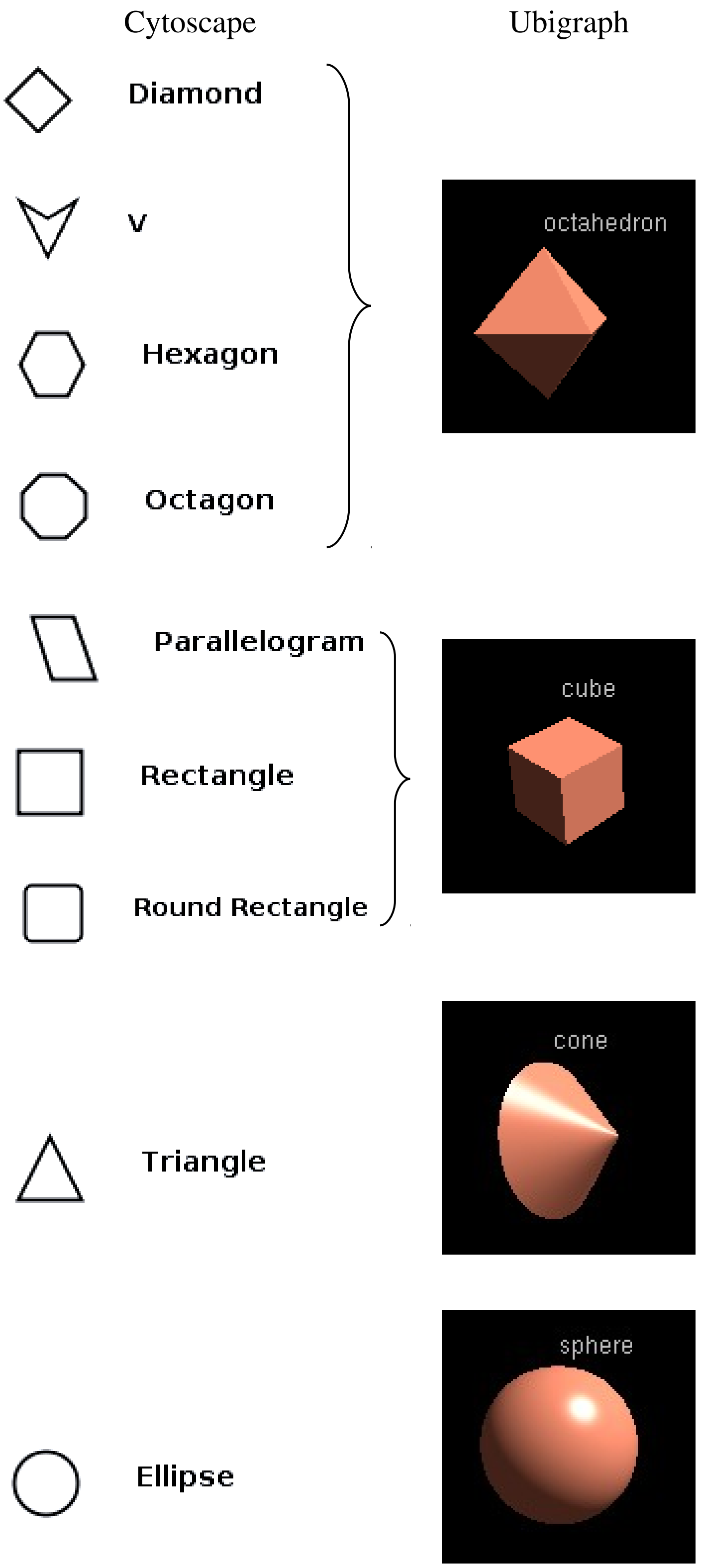

The default shape in Ubigraph is cube. If all nodes in cytoscape network view are round, they are converted to cube automatically after conversion for improving performance.

Search node/edge

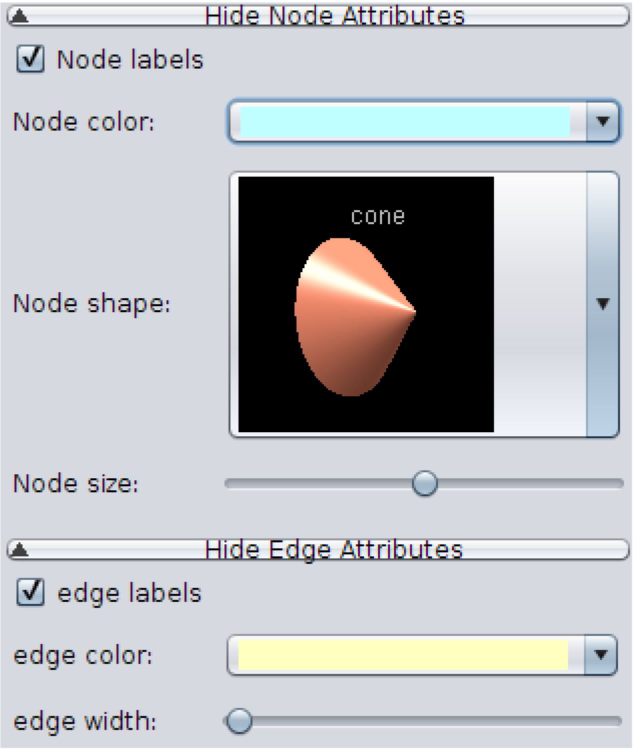

Type node/edge label in the search box, and it will try to auto-complete it. There is an option to specify which term to search beside the box. The selected node/edge will be highlighted with a label beside it in the Ubigraph window.

Selection

Double click a node in Ubigraph will return the node label in an **"node attribute browser"** in the Cytoscape panel below. Change the appearance of the selected node/edges or add/delete them in Cytoscape will also be reflected in the Ubigraph 3D renderer.

Node/Edge Attributes

Node/Edge attributes such as label, color, shape, size can be set in each panel. You can collapse the panel **"Show Node/Edge Attributes"** and use the buttons and sliders in the panel. The Node/Edge attributes changes will be applied to all node/edges whenever there is no node/edge selected in Cytoscape. If there is node/edge selected in Cytoscape, only the selected ones will be changed.

Visualize Expression Data

Expression data can be loaded in Cytoscape expression matrix data format http://wiki.cytoscape.org/Cytoscape_User_Manual/Expression_Data . Load the data either from File->Import->Attribute/Expression Matrix or from the

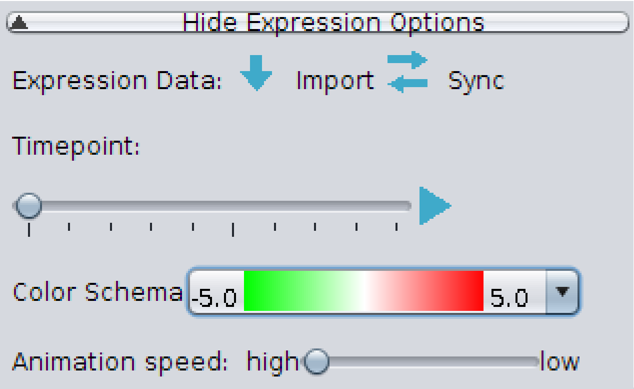

Import button. If data are loaded before the 3D conversion, use the Sync button to synchronize expression matrix with the 3D network. The nodes are rendered according to a gradient color schema, in which the positive values are rendered in red, and negative values are rendered in green. The label Timepoint shows the condition in each time-point. By pressing the Play/Pause button, the 3D graph replays through all conditions automaticly.

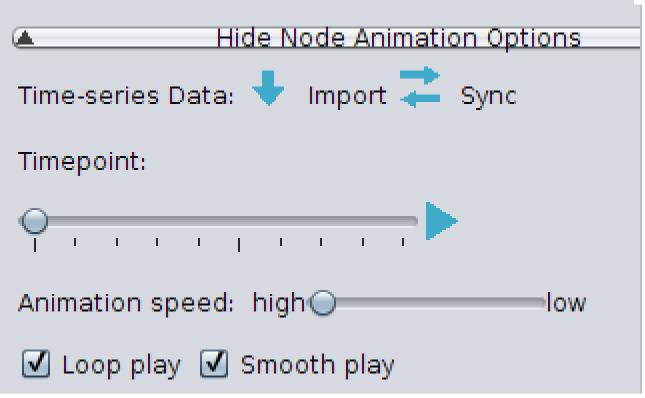

Node/edge animation

Node/edge can indicate relative values according to their size/width, which is usefully in visualizing time-series data, such as metabolic

flux. The data should be prepared as tab-delimited format, see the following example. Each row has the label of the node/edge, and each column are specified with a condition.

Pathway	4h	8h	16h
G6P (pd) Glucose	1	2	3
G1P (pp) G6P	2	1	3
G6P (pp) F6P	3	2	1

Load the data using **Import** button, If data are loaded before the 3D conversion, use the **Sync** button to synchronize data with the 3D network. The values relative to the nodes/edges are rendered in different node size/edge width.

Multiple network animation

3DScapeCS can join a series of networks into one motion network if the networks share nodes with the same label. To create a motion network, use the button "Order networks" in the "N**etwork Animation"** panel. In the dialog popped up, you can add the networks you would like to use. A motion network is created hereafter. You can use the Play/Pause button to replay each network. The animation speed control is available beneath the play button. By selecting the "**Fixed Play**", 3DScapeCS will only render nodes/edges invisible from one motion frame to another, instead of adding/removing them from the network. By selecting the "**Accumulative Play**", 3DScapeCS will only add nodes/edges from one motion frame to the next frame, but do not remove them even they are not presented in the following network. The label showing the name of experiment in each time points will be rendered in different colors as well as the node/edges appeared with each time points.

Comparison between fixed and default visualization

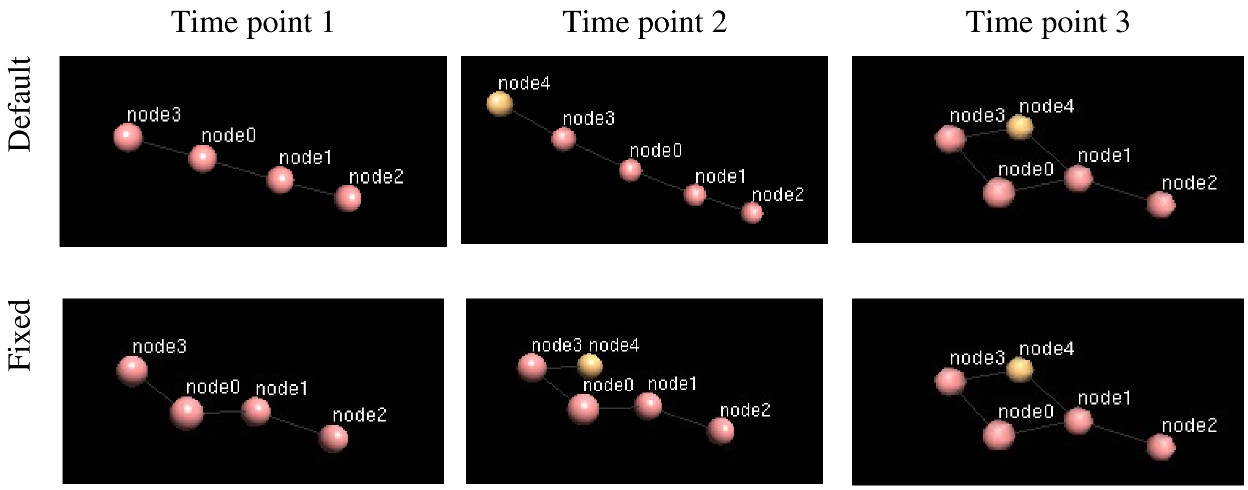

Parallel Monitor Support

3DScapeCS use XML-RPC protocol to communicate with each ubigraph_server. Therefore you can open several ubigraph_server on different computers, just to make sure the clients are in the same network. The default client is your local host with an IP address 127.0.0.1. You can add another network client using **Add** button. In the dialog popped up, you input the IP address of destinate network client. To remove a network client from the panel, use press the **Remove** button. The **Latency slider** can be use for specify different time points for each client whenever playing through multiple time point animation, such as expression data.

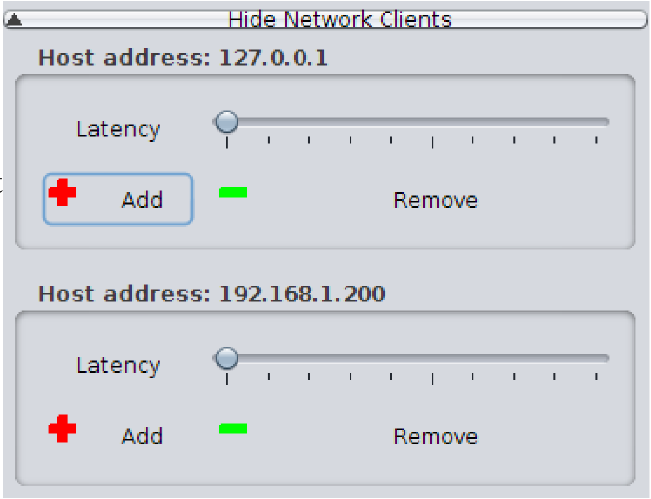

Multiple monitor visualizaiton

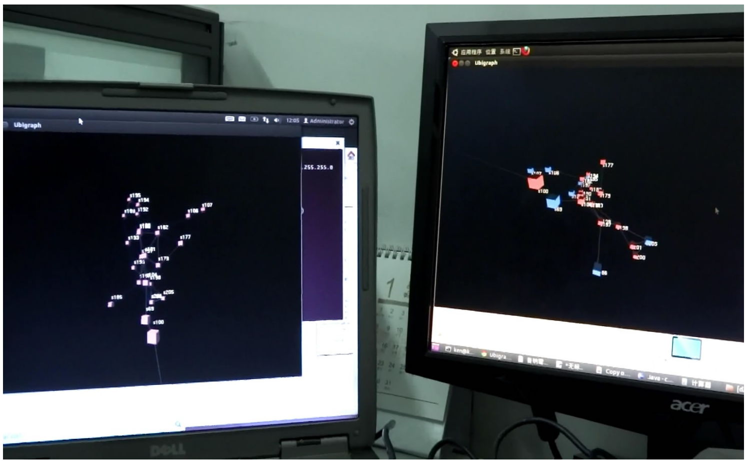

Ubigraph Operations

Read the following operation instructions found in Ubigraph document.

Key(s)	Function
ESC	Exit full-screen mode
↑ and ↓	Zoom in/out
!	Zoom way out
@	Zoom way in
← and →	Start/increase/stop y-axis rotation
u, d	Start/increase/stop z-axis rotation
r	Reset vertices to random positions
+,-	Increase/decrease time step
h	Toggle Runge-Kutta/Euler step

p	Toggle serial/parallel
f,F	Decrease/increase friction
v	Toggle draw vertices
s	Toggle draw strain
S	Show performance stats

Frequently Asked Questions (FAQs)

Q: Why Ubigraph freeze when I add lots of nodes/edges?

A: It seems to be a problem with the 64-bit release. Try 32-bit release instead if you encounter this. See this post http://stackoverflow.com/questions/5557100/free-alternative-to-ubigraph for detail.

Q: Why I saw "libstdc++.so.6: version `GLIBCXX_3.4.9` not found ..." when I started Ubigraph.

A: You may need to remake a soft link to libstdc++.so.6.0.10 using the following command:

```
ln -s /urs/lib/libstdc++.so.6.0.10 /urs/lib/libstdc++.so.6
```

Q: Why Ubigraph freezes when I converted a graph/drag time-point slider/change node attributes?

A: Ubigraph sometimes freezes when there is heavy computational load. One solution is to terminate it using “Ctrl+C” in the terminal and then start it again. Also remind you that Ubigraph window should be kept in background when there is heavy computational load.

Copyright

THE PROGRAM IS PROVIDED "AS IS" WITHOUT WARRANTY OF ANY KIND, EITHER EXPRESS OR IMPLIED, INCLUDING, BUT NOT LIMITED TO WARRANTIES OF MERCHANTABILITY OR FITNESS FOR A PARTICULAR PURPOSE. IN NO EVENT WILL THE AUTHOR or AUTHORS BE LIABLE TO YOU FOR ANY DAMAGES, INCLUDING INCIDENTAL OR CONSEQUENTIAL DAMAGES, ARISING OUT OF THE USE OF THE PROGRAM, EVEN IF ADVISED OF THE POSSIBILITY OF SUCH DAMAGES.

YOU ACKNOWLEDGE THAT YOU HAVE READ THIS LICENSE, UNDERSTAND IT AND AGREE TO BE BOUND BY ITS TERMS AS THE COMPLETE AND EXCLUSIVE STATEMENT OF THE AGREEMENT BETWEEN US, SUPERSEDING ANY PROPOSAL OR PRIOR AGREEMENT, ORAL OR WRITTEN, AND ANY OTHER COMMUNICATIONS BETWEEN US RELATING TO THE SUBJECT MATTER OF THIS LICENSE.

3DScapeCS is free software; you can redistribute it and/or modify it under the terms of the GNU Lesser General Public License as published by the Free Software Foundation; either version 2.1 of the License, or (at your option) any later version.. You should have received a copy of the GNU Lesser General Public License, The program is distributed in the hope that it will be useful, but WITHOUT ANY WARRANTY; without even the implied warranty of MERCHANTABILITY or FITNESS FOR A PARTICULAR PURPOSE. See the GNU Lesser General Public License for more details.

You should have received a copy of the GNU Lesser General Public License along with this library;

if not, you can get a copy from http://www.gnu.org/licenses/lgpl.html or write to the Free Software Foundation, Inc., 59 Temple Place, Suite 330, Boston, MA 02111-1307 USA

Contact

For more questions or bug report, please e-mail to qwang.big@gmail.com
